# Major Molecular Response Achievement in CML Patients Can Be Predicted by *BCR-ABL1/ABL1* or *BCR-ABL1/GUS* Ratio at an Earlier Time Point of Follow-Up than Currently Recommended

**DOI:** 10.1371/journal.pone.0106250

**Published:** 2014-09-09

**Authors:** Sarah Huet, Pascale Cony-Makhoul, Maël Heiblig, Isabelle Tigaud, Sophie Gazzo, Amine Belhabri, Denis Souche, Mauricette Michallet, Jean-Pierre Magaud, Sandrine Hayette, Franck Nicolini

**Affiliations:** 1 Laboratoire d'hématologie, Centre de Biologie Sud, Centre Hospitalier Lyon Sud, Pierre-Benite, France; 2 Unité Mixte de Recherche 5239 Centre National de la Recherche Scientifique, Faculté de médecine Lyon Sud, Oullins, France; 3 Service clinique d'hématologie, Centre Hospitalier d'Annecy, Pringy, France; 4 Service clinique d'hématologie 1G, Centre Hospitalier Lyon Sud, Pierre-Benite, France; 5 Service clinique d'hématologie, Centre Régional de Lutte Contre le Cancer Léon Bérard, Lyon, France; UT MD Anderson Cancer Center, United States of America

## Abstract

Recent studies demonstrate that early molecular response to tyrosine-kinase inhibitors is strongly predictive of outcome in chronic myeloid leukemia patients and that early response landmarks may identify patients at higher risk for transformation who would benefit from an early switch to second-line therapy. In this study, we evaluated the ability of the control gene *GUS* to identify relevant thresholds for known therapeutic decision levels (*BCR-ABL1/ABL1^IS^*  = 10% and 0.1%). We then defined the most relevant cut-offs for early molecular response markers (transcript level at 3 months, halving time and log reduction between diagnosis and 3 months of treatment) using *GUS* or *ABL1*. We demonstrated that, although both control genes could be used (in an equivalent way) to accurately assess early molecular response, the *BCR-ABL1/GUS* level at diagnosis is impacted by the higher *GUS* copy number over-expressed in CML cells, thus negatively impacting its ability to completely replace *ABL1* at diagnosis. Furthermore, we pointed out, for the first time, that it would be helpful to monitor *BCR-ABL1* levels at an earlier time point than that currently performed, in order to assess response to first-line tyrosine-kinase inhibitors and consider a potential switch of therapy as early as possible. We evaluated this optimal time point as being 19 days after the start of treatment in our cohort.

## Introduction

The European Leukemia Network (ELN) recommendations for the management of chronic myeloid leukemia (CML) patients define optimal response, warning or failure according to cytogenetic and/or molecular criteria obtained at 3, 6 and 12 months on tyrosine-kinase inhibitors (TKI) therapy [1] and optimal response is associated with best long-term outcome. Indeed, several studies have highlighted that the achievement of early molecular and cytogenetic responses on TKI was predictive of long term event-free survival (EFS), treatment-free survival (TFS) and overall survival (OS) and that this ability to predict outcome is observed for all TKI although with different kinetics [2]–[11]. Marin et al. reported that *BCR-ABL1^IS^* levels at 3 and 6 months on Imatinib were significantly correlated with 8-year progression-free survival (PFS) and OS [12], and Hanfstein et al. proposed *BCR-ABL1^IS^* levels of 10% at 3 months and 1% at 6 months as clinically important thresholds correlated with 5-year PFS and OS [3]. Therefore, early molecular response (EMR) to TKI is currently identified as being one of the most important prognostic factors, and early response landmarks may identify patients at higher risk for transformation and poor outcome, who may benefit from alternative treatments in order to improve response and thereby minimize exposure to risk over time.

Is was also demonstrated that EMR at 3 and 6 months correlates with future major molecular response (MMR) and deep molecular response (ie, molecular response ≥4.5-log reduction [MR^4.5^] and beyond) [5], [9], [10], [13]. Although the prognostic significance of achieving MMR at 12 or 18 months has been controversial in the past [14], [15], Hughes et al. showed that patients who achieved MMR by 12 and 18 months while on Imatinib therapy had significantly improved 7-year EFS and PFS rates [4], thus demonstrating a strong association between MMR achievement and long-term clinical outcome. Moreover, reaching the 12-month MMR still represents an ELN criterion of optimal response and should be a main goal in the management of the patient.

**Figure 1:**
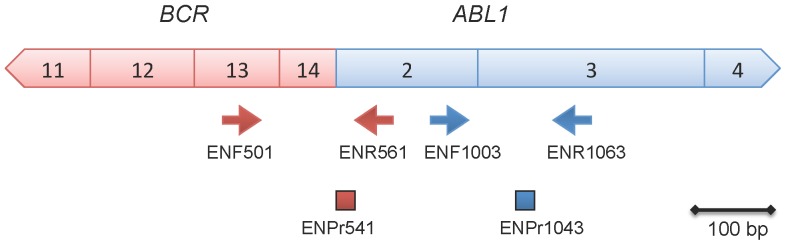
Quantification bias in the assessment of *BCR-ABL1* transcript levels by using *ABL1* as control gene. Localization of primers (arrows) and probes (rectangles) used for the relative quantification of the *M-BCR-ABL1* transcript according to the EAC protocol [16], [17] are represented. Boxes represented the exon number. Red signs indicate primers and probe relative to the fusion transcript, blue signs to the control gene *ABL1*. The *ABL1* primers localization explains both amplification of *ABL1* and *BCR-ABL1* during the Q-RT-PCR reaction. ENF = forward primer, ENPr = TaqMan reverse probe, ENR = reverse primer.

This growing interest in the assessment of EMR led to reconsider the use of *ABL1* as control gene (CG) when quantifying the *BCR-ABL1* transcript. This CG was selected by a Europe Against Cancer (EAC) study group [16], [17] but has the disadvantage of inducing a quantification bias in determining *BCR-ABL1* transcript levels. Indeed the location of *ABL1* primers leads to the simultaneous amplification of the non-translocated allele of *ABL1* and the fusion gene *BCR-ABL1*, then quantifying total-*ABL1* (figure 1). This may lead to underestimation of *BCR-ABL1/ABL1*% when *BCR-ABL1* expression is high. Although *BCR-ABL1* level at diagnosis has not been clearly identified as being of prognostic significance by itself and has been cause for controversy [18], [19], it allows (at least) the assessment of early transcript kinetics (for example between diagnosis and 3–6 months of treatment), which recently arose as an important parameter of the EMR [1], [19], [20]. Thus, the fact that *BCR-ABL1* transcript level at diagnosis should be measured as accurately as possible encouraged efforts to find another CG. Accordingly, the *GUS* gene, encoding for beta-glucuronidase, previously identified as a suitable CG in CML [16], attracted interest for quantifying *BCR-ABL1* and assessing EMR to TKI [18], [19].

Our study intended (i) to assess the ability of *GUS* to normalize the *BCR-ABL1* level without technical bias at diagnosis and validate its potential predictive value at this time (ii) to evaluate its ability to accurately define the relevant thresholds for known therapeutic decision levels (10% and major molecular response MMR: 0.1%) and (iii) to compare different markers of EMR (IS cut off, halving time and fold reduction of transcript ratios) using *GUS* and *ABL1* to predict the achievement of MMR after 12 months of TKI in a series of front line therapy patients. We describe the optimal cut-offs for the different markers to predict the 12-month MMR achievement with confirmation in a validation cohort. We highlight that it could be appropriate to add a new, earlier time point to patient monitoring earlier than those currently recommended in clinical practice in order to identify patients likely to benefit from an early switch of TKI.

## Subjects and Methods

### 1. Patients and samples

CML patients included in this retrospective analysis were diagnosed and monitored on TKI in our institutions since August 2000 and the blood samples were collected between August 2000 and January 2013. Patients gave written informed consent for their data to be used in this analysis and the procedures followed were in accordance to the Helsinki declaration as revised in 2008. Samples were stored in the Biological Resource Center Bank according to the French “Comité de Protection des Personnes” specifications. The review board protocol of the Hospices Civils de Lyon approved this study. To address the main questions of the study, samples were collected as follows:

First, we focused on the transcript values obtained by using *GUS* as a CG. Therefore, we studied 3 groups of samples according to their transcript values: the first group comprised diagnostic samples (n = 124), the second group comprised follow-up samples with a *BCR-ABL1/ABL1^IS^* ratio between 6 and 14% (n = 18) and the third group consisted of samples at the MMR IS threshold of 0.1% (n = 42). We were particularly interested in these transcript values since (i) diagnosis is highly impacted by the quantification bias but is crucial to assess the EMR (for example between diagnosis and 3–6 months of treatment) and (ii) they represent the relevant thresholds for known therapeutic decision levels (10% and major molecular response MMR: 0.1%).
We then went on to evaluate the prognostic value (probability of obtaining MMR one year after diagnosis) of different parameters described in the literature for assessing the early molecular response (EMR) value. We then selected patients with available samples at diagnosis and after 3 months of TKI therapy. Of the 124 patients obtained at diagnosis, patients with the following criteria were included: (1) TKI therapy started less than 60 days after diagnosis (median, 29 days; range, 0–58 days), (2) the patient should have received TKI between 60 and 120 days before the follow-up so-called “3-month” sample (median, 90 days; range, 62–117 days; 4 of them received between 60 and 75 days of TKI) and (3) molecular follow-up assessed for at least 1 year after diagnosis in order to evaluate the achievement of MMR (median follow-up 3.9 years; range 1.1–9.7 years). One patient who simultaneously presented another haematological malignancy and died before 1 year of treatment was excluded. Patient characteristics at diagnosis are listed in table 1. Of the 84 patients who met the inclusion criteria, 55 were treated with Imatinib and 29 received second-generation TKI (Nilotinib, n = 12 or Dasatinib, n = 17). Treatment interruption and/or switching was documented for 7 patients on Imatinib (temporary interruption, n = 4 and switch to Nilotinib, n = 3) and 2 patients on Nilotinib (1 temporary interruption and 1 switch to Imatinib then Dasatinib because of blast crisis).
We validated our results in a supplemental cohort of patients (n = 58) selected according to the same criteria. Forty patients were on Imatinib as first-line treatment, 9 on Dasatinib and 9 on Nilotinib. Treatment interruption and/or switching was documented for 6 patients on Imatinib (switch to Nilotinib, n = 3; switch to Dasatinib, n = 3).

**Table 1:** Patient Characteristics (N = 84).

Characteristics	No.	*%*
**Age, years**		
Median	58	
Range	20–89	
**Sex**		
Male	58	*69*
Female	26	*31*
**Sokal risk group** *		
Low	19	*24*
Intermediate	34	*43*
High	23	*29*
Accelerated phase at diagnosis	3	*4*
**Chromosomal abnormalities in addition to the Philadelphia chromosome** **	8	*10*

*We could not calculate Sokal score in 5 patients because of missing data.

**Information relative to chromosomal abnormalities was not available for 2 patients.

### 2. Molecular analysis

RNA was extracted from peripheral blood sample and reverse transcription (RT) and Quantitative Real-time-PCR (RQ-PCR) to amplify *BCR-ABL1* fusion transcript *ABL1* and *GUS* were performed according to standardised EAC protocols previously described [16], [17]. Only patients expressing typical *BCR-ABL1* transcripts (b2a2 or b3a2) were considered. The final results are expressed as *BCR-ABL1*/*ABL1* ratios in percent according to the international scale IS (i.e. the conversion factor was applied for raw ratios ≤10%, and not for raw values >10%) as it was previously recommended [21], [22] or as *BCR-ABL1*/*GUS*, without transformation of the original values since no conversion factor was defined for this CG. Samples with copy numbers that were too low (CN<10,000) for either *ABL1* or *GUS* were presumably degraded samples (according to the GBMHM [Groupe des Biologistes Moléculaires de Hémopathies Malignes] recommendation guidelines: https://sites.google.com/site/gbmhmassociation/) and were excluded.

### 3. Statistical and result analysis

The alignment between both methods using (*BCR-ABL1*/*ABL1)^IS^* and *BCR-ABL1*/*GUS* values was performed by setting a new conversion factor (CF), following the procedure that has been used previously to set the international conversion factor IS [21]. To determine this new CF, each measurement generated by using *ABL1* as CG was compared with that generated with *GUS* for the same sample. The bias between both measurements reflects the tendency of a method using one given CG to exceed the method using another CG. It was calculated as the difference between both measurements: bias  =  log(*BCR-ABL1/ABL1^IS^*) – log(*BCR-ABL1/GUS*) for each sample. The CF is calculated as the antilog of the mean bias between measurements with both methods.

Three markers of EMR were used: halving time, transcript level at 3 months and log reduction between diagnosis and 3 months on TKI. The halving time defines the number of days over which the *BCR-ABL1* transcript value shows a 2-fold decrease [20], [23]. According to Branford et al, the following formula was used: halving time = ln2*d/[ln(a)-ln(b)] where (a) is the transcript value at diagnosis, (b) the transcript value of the 3-month follow-up and (d) the number of days between both measurements. The log reduction in transcript level is another measurement of early molecular response [19]. This was defined as log(Transcript level at diagnosis/Transcript level at 3 months). All the analyses were performed on an intention-to-treat basis. Groups were compared using the ANOVA or the Kruskal-Wallis test adjusted for multiple comparisons when relevant. The Fisher Exact test and χ^2^ test were used to compare frequencies. Correlations between different variables were investigated using the Pearson test.

## Results and Discussion

### 1. The use of *GUS* as CG requires the application of a conversion factor

In the first group of patients, the *BCR-ABL1*/*ABL1* median ratio at diagnosis was 69.3% (range: 25.0–121.0%) whereas the *BCR-ABL1*/*GUS* median ratio was 15.1% (range: 8.3–51.1%).

In the second group (patients chosen for their transcript *BCR-ABL1^IS^* values comprised between 6% and 14%), the median ratios of these transcripts were 11.1% for *BCR-ABL1*/*ABL1* (range: 6.7–13.8%) and 3.1% (range: 2.2–5.8%) for *BCR-ABL1/GUS*.

In the third group (patients with transcript levels at 0.1%), the median ratios were 0.089% for *BCR-ABL1*/*ABL1* (range: 0.047–0.183%) and 0.035% (range: 0.016–0.072%) for *BCR-ABL1/GUS*. As samples were chosen according to their transcript value of 0.1% when analysed in laboratory routine practice, and that all measurement were performed again simultaneously for this study, ratios vary slightly in this range of values. Therefore, we will refer to this group of samples as “MMR-related samples” later in this study.

As expected, *BCR-ABL1*/*ABL1^IS^* and *BCR-ABL1*/*GUS* values showed a direct correlation (ρ = 0.762; p<0.001).

**Figure 2:**
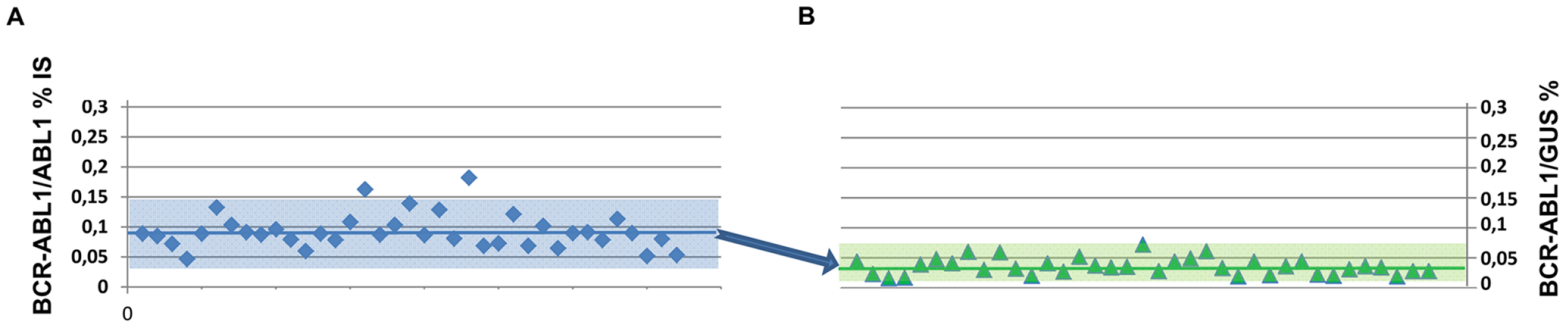
The use of *GUS* as control gene would need the introduction of a conversion factor. The conversion from *BCR-ABL1*/*ABL1* to *BCR-ABL1*/*GUS* values would need the introduction of an additional conversion factor to define the major thresholds used in the clinics for the MRD monitoring, as exemplified for the MMR threshold. (**a**) Graph A plots the (*BCR-ABL1/ABL1)^IS^* % value of each patient close to the MMR threshold. The mean of 42 samples was 0.092%, 2 s.d. range 0.038–0.147%. (**b**) Graph B plots the *BCR-ABL1/GUS* % value of the same samples. The mean was 0.036% and 2 s.d. range 0.010–0.062%.

These results indicate that the conversion from *BCR-ABL1*/*ABL1* to *BCR-ABL1*/*GUS* values would require the introduction of a further conversion factor in order to adapt them to the decision thresholds currently used for MRD monitoring. This conversion is exemplified in figure 2 for the MMR threshold, for which the transcript ratio of 0.1% using *ABL1* corresponds to a ratio of 0.036% using *GUS*.

**Figure 3:**
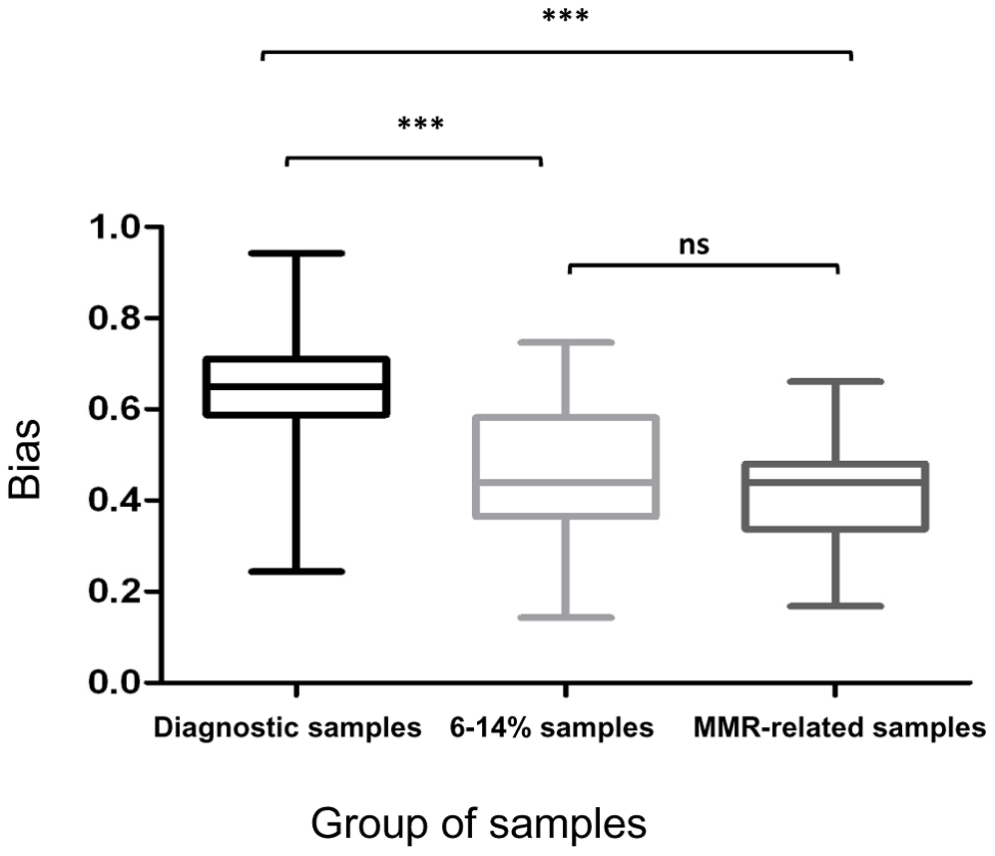
Box plot of the bias between *(BCR-ABL1/ABL1)^IS^* and *BCR-ABL1/GUS* measurement in the 3 groups of samples. The bias (leading to conversion factor calculation) was compared between 3 groups of samples: diagnostic samples, samples with 6–14% and 0.1% transcript levels. It is significantly higher in the group of diagnostic samples (*** represents p-value<0.001; ns, non-significant).

The bias between measurements using *ABL1* or *GUS* was calculated for each sample. In diagnostic samples, the mean bias was 0.65 (standard deviation SD: 0.12) whereas it valued at 0.45 (SD: 0.16) in samples with a 6–14% *BCR-ABL1*/*ABL1* ratio and was somewhat similar, 0.42 (SD: 0.12) in the third group. The CF was then calculated as the antilog of the mean bias and valued at 4.45, 2.84, and 2.65 in the three groups of samples, respectively (table 2). A CF of 2.14 was reported by Hanfstein et al [19]. Nevertheless, we observed that the mean bias (and therefore the CF) differed within the different groups of samples (table 2 and figure 3). In particular, it was significantly different in the group of diagnostic samples (p<0.001, adjusted for multiple comparison test) whereas it did not differ significantly between the two other groups. Therefore, the conversion factor from *BCR-ABL1*/*ABL1* to *BCR-ABL1*/*GUS* is not constant within the different disease times and contrary to what was expected, is more pronounced at diagnosis implying that *GUS* is not a better GC than *ABL1* at diagnosis.

**Table 2:** Summary of the results obtained in the 3 groups of samples.

	Diagnosis samples	6–14% samples	0,1% samples	p
n	124	18	42	/
***BCR-ABL1*** **/** ***ABL1^IS^*** ** ratio (%)**				
median	69.3	11.1	0.089	/
range	25.0–121.0	6.7–13.8	0.047–0.183	
***BCR-ABL1*** **/** ***GUS*** ** ratio (%)**				
median	15.1	3.1	0.035	/
range	8.3–51.1	2.2–5.8	0.016–0.072	
**Bias**				
mean	0.65	0.45	0.42	<0.001
standard deviation	0.12	0.16	0.12	
antilog of bias = Conversion Factor	4.45	2.84	2.65	
***GUS*** ** copy number**				
mean	290,800	117,200	110,900	<0.001
standard deviation	164,400	46,700	28,900	
***ABL1*** ** copy number**				
mean	66,500	37,900	33,700	<0.001
standard deviation	49,300	16,700	9,200	

### 2. *GUS* is overexpressed at diagnosis

**Figure 4:**
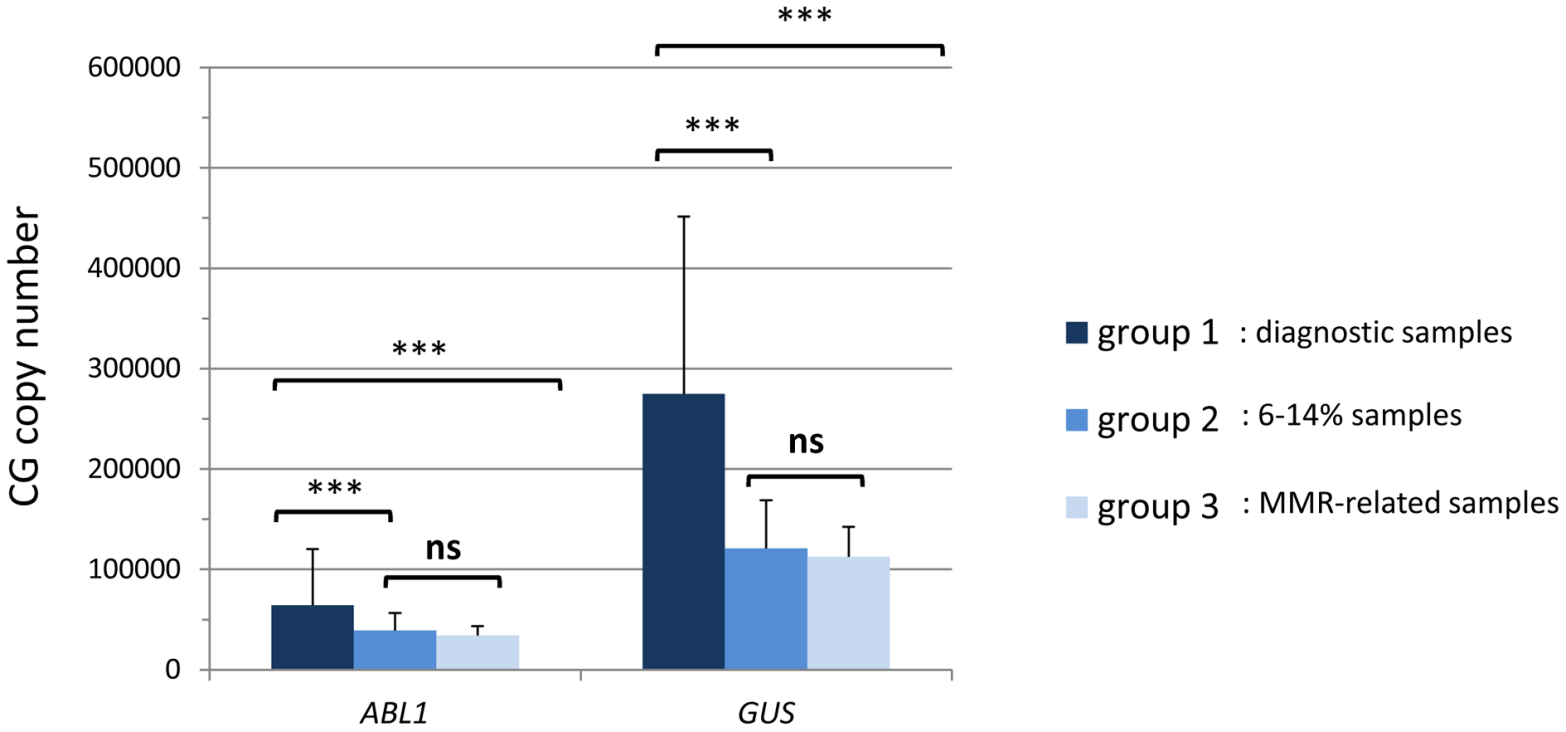
Mean copy number of both control genes for the 3 groups of samples. *GUS* and *ABL1* copy number is significantly higher at diagnosis (*** represents p-value<0.001; ns, non-significant).

This difference in CF values during the course of the disease was due to the overexpression of *GUS* at diagnosis, with *GUS* mean copy number of 290 800, 117 200 and 110 900 in the 3 groups, respectively (p<0.001). Thus the higher copy number of *GUS* at diagnosis is responsible for the higher CF. Although *ABL1* is also overestimated at diagnosis due to the quantification test bias (p<0.001), *GUS* expression varies at least in the same range of values as *ABL1* (mean copy number: 66 500, 37 900 and 33 700, respectively) (Figure 4). The values of transcripts, gene copy number and conversion factor for the 3 groups are summarized in table 2.

This higher copy number of *GUS* at diagnosis is not due to the higher number of cells at this point in the disease, since the reverse-transcription protocol is designed to ensure that the same amounts of RNA are used whatever the sample is (MRD or diagnosis) [16], [17].

**Figure 5:**
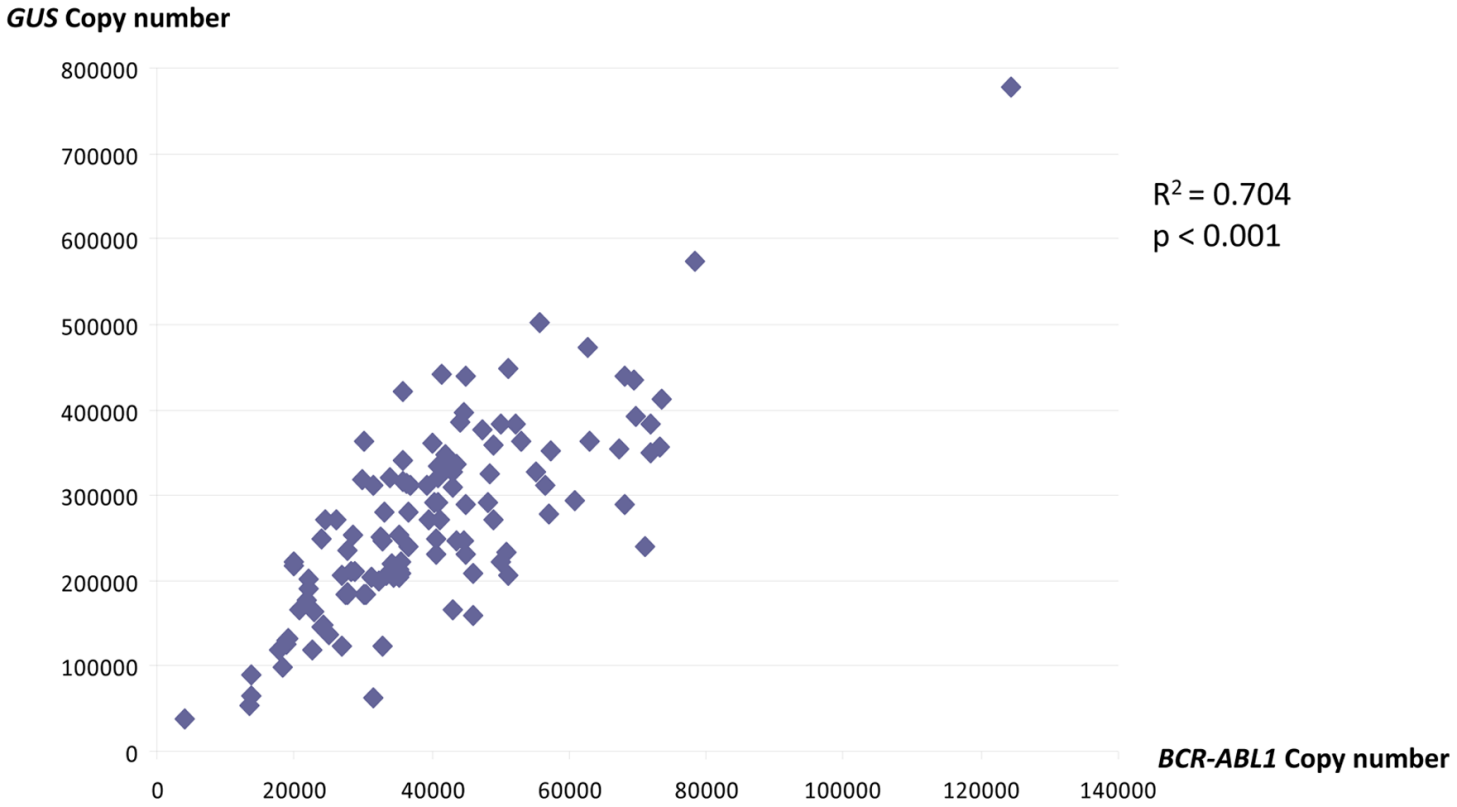
The copy number of *GUS* and *BCR-ABL1* are highly correlated. This supports the hypothesis that *GUS* may be overexpressed in leukemic cells compared to normal cells.

It was previously reported that *GUS* was overexpressed at CML diagnosis compared to healthy donors [16]. We thus hypothesized that *GUS* may be overexpressed in leukemic cells compared to normal cells and that *GUS* quantification may be correlated to the *BCR-ABL1* transcript level. Indeed, a significant correlation was found between *GUS* and *BCR-ABL1* copy number (R^2^ = 0.704; p<0.001, figure 5).

This overexpression of *GUS,* impacts the *BCR-ABL1*/*GUS* ratio at diagnosis and warrants the use of a unique conversion factor for all disease levels. Nevertheless, the number of copies of *GUS* did not differ significantly between the 6–14% samples and MMR-related samples, nor did the *ABL1* copy number. Therefore, the quantification bias observed for diagnostic samples does not seem to impact the values under 14%.

### 3. The transcript level at diagnosis does not impact on the achievement of MMR

In order to determine whether the transcript level at diagnosis could be used as a prognostic indicator for disease evolution, we compared patients who achieved the MMR threshold 1 year after TKI introduction (n = 70) and those who did not (n = 54). The *BCR-ABL1*/*GUS* median ratio at diagnosis was 13.78% in the first group and 15.96% in the second, and it did not differ significantly between both groups; neither did the *BCR-ABL1*/*ABL1* median ratio (69.3% in both groups). The sub-group analysis for patients receiving TKI 1^st^ or 2^nd^ generation did not show any other difference for the *BCR-ABL1*/*GUS* or *BCR-ABL1*/*ABL1* ratios. Besides, no prognostic cut-off could be identified for either *BCR-ABL1*/*GUS* or *BCR-ABL1*/*ABL1* at diagnosis. Therefore, the transcript level at this time, whatever CG is used, does not predict the achievement of MMR after one year of treatment. Another group reported that elevated *BCR-ABL1/GUS* at diagnosis correlated with inferior probabilities of optimal response [18]. We could not confirm these findings since we did not identify any prognostic cut-off, which is in accordance with a recent report assessing the absence of prognostic significance of transcript level at diagnosis on OS and PFS [19].

### 4. The transcript kinetics between diagnosis and 3 months of treatment predicts reaching the MMR levels at 12 months

The median *BCR-ABL1^IS^* level in this 84-patient cohort was 69.6% (range, 25.0–95.7%) at diagnosis and 0.98% (range, 0.01–36.9%) at 3 months. We used a receiver operating characteristic (ROC) curve to identify the optimal cut-off for the different variables tested that would allow us to classify the patients as presumably achieving or not the MMR at 12 months with maximal sensitivity and specificity (table 3).

**Table 3:** Prediction of MMR achievement at 12 months with the use of different EMR markers.

		Analysis with *(BCR-ABL1/ABL1)^IS^*					Analysis with *BCR-ABL1/GUS*				
EMR marker		Cut-off	No. of patients	OR	95% CI	p	Cut-off	No. of patients	RR	95% CI	p
**Transcript level at 3 months (%)**											
TKI1											
Low risk		≤0.99	28	23.81	5.45–104.1	<0.001	≤0.36	27	20.00	4.67–85.60	<0.001
High risk		>0.99	27				>0.36	28			
TKI2											
Low risk		≤0.99	16	16.50	2.51–108.6	0.003	≤0.36	14	6.88	1.35–35.07	0.027
High risk		>0.99	13				>0.36	15			
All TKI											
Low risk		≤0.99	44	18.21	6.08–54.54	<0.001	≤0.36	41	12.55	4.38–35.92	<0.001
High risk		>0.99	40				>0.36	43			
**Halving time (days)**											
TKI1											
Low risk		≤19	31	33.25	7.39–149.6	<0.001	≤21	30	36.00	7.69–168.6	<0.001
High risk		>19	24				>21	25			
TKI2											
Low risk		≤19	15	10.08	1.82–56.02	0.009	≤21	14	6.88	1.35–35.07	0.027
High risk		>19	14				>21	15			
All TKI											
Low risk		≤19	46	20.89	6.81–64.08	<0.001	≤21	44	18.21	6.08–54.54	<0.001
High risk		>19	38				>21	40			
**Log reduction**											
TKI1											
Low risk		≤1.70	31	25.65	6.08–108.3	<0.001	≤1,45	32	19.44	4.91–76.96	<0.001
High risk		>1.70	24				>1,45	23			
TKI2											
Low risk		≤1.70	16	16.50	2.51–108.6	0.003	≤1,45	16	16.50	2.51–108.6	0.003
High risk		>1.70	13				>1,45	13			
All TKI											
Low risk		≤1.70	47	20.89	6.81–64.08	<0.001	≤1,45	48	17.95	5.98–53.86	<0.001
High risk		>1.70	37				>1,45	36			

We compared the ability 3 early molecular response (EMR) markers recently described (transcript level at 3 months, Halving time and transcript log reduction) to accurately predict the achievement of major molecular response (MMR) after 12 months of treatment, either with the use of *ABL1* or *GUS* as control gene. Optimal cut-off for each marker was identified by using receiver operating characteristic (ROC) curve. Relative risks are expressed as odds ratios (OR) with 95% confidence interval (95% CI).

#### Transcript level at 3 months

It was recently demonstrated that the persistence of *BCR-ABL1^IS^* transcript levels above 10% at 3 months identified a group of high-risk patients that would benefit from treatment optimization [3], [12]. Similarly, Marin et al. found that patients with a transcript value ≤2.81% had a significantly higher rate of MMR achievement during an 8-year follow-up [12]. Branford et al. reported a cut-off level for MMR achievement of 1.4% [20]. We tested, in our cohort, whether another cut-off point could predict long-term achievement of MMR at 12 months. Patients with *BCR-ABL1^IS^* transcript levels >0.99% at 3 months had less chance of attaining the 1-year MMR threshold (18% vs. 84%, p<0.001). These results were concordant with recent reports regarding the 3-month landmark analysis predictive of 1-year MMR [3], including for separate analysis of patients on TKI 1^st^ and 2^nd^ generation [5]. The use of *BCR-ABL1/GUS* levels with a cut-off at 0.36% (corresponding to the 0.99% *BCR-ABL1^IS^* corrected by the conversion factor of 2.65 defined for this MRD level) led to the same conclusions (p<0.001), for both generation of TKI.

#### Halving time

The halving time defines the number of days over which the *BCR-ABL1* transcript value shows a 2-fold decrease [20], [23]. The median halving time was 18 days (range, 9–202 days) and was similar between Imatinib (18 days) and 2^nd^ generation TKI (19 days). Using *(BCR-ABL1/ABL1) ^IS^*, we found that patients with a halving time ≤19 days (n = 46) had a superior rate of MMR achievement at 12 months than patients with halving time >19 days (n = 38) (85% vs. 18%, p<0.001). Similarly to the predictive cut-off for transcript level at 3 months which varies according to the end-point of the study, our halving time cut-off value for 1-year MMR is lower than the 90-day cut-off used by Branford et al. to predict survival [20], but confirms that the transcript kinetics also represents an important information to predict the MMR achievement. Using *BCR-ABL1/GUS*, we found that the cut-off of 21 days separated both groups with the maximal accuracy (22.5% vs. 84%, p<0.001). The same results were obtained by TKI sub-group analysis.

#### Log reduction between diagnosis and 3 months

Similarly, the reduction of the transcript *BCR-ABL1*/*GUS* ratio between diagnosis and 3 months of treatment was reported as a predictor of survival [3], [19]. The median reduction in our cohort was a 43-time decrease, corresponding to a 1.63 log reduction, and we identified the value of 1.45 log reduction at 3 months as the best predictive cut-off for MMR achievement. Eighty-one % of patients who obtained a log reduction >1.45 achieved the MMR threshold at 12 months, vs. 19% of patients with a log reduction ≤1.45 (p<0.001). By using the *(BCR-ABL1/ABL1) ^IS^* ratio, the median log reduction was 1.86 and a cut-off of 1.70 log reduction allowed the best discrimination for MMR achievement (83% vs. 19%, p<0.001). The same results were obtained by TKI sub-group analysis. Of note, all the patients with a *BCR-ABL1^IS^* transcript level ≤0.99% at 3 months also obtained a log reduction >1.70.

**Figure 6:**
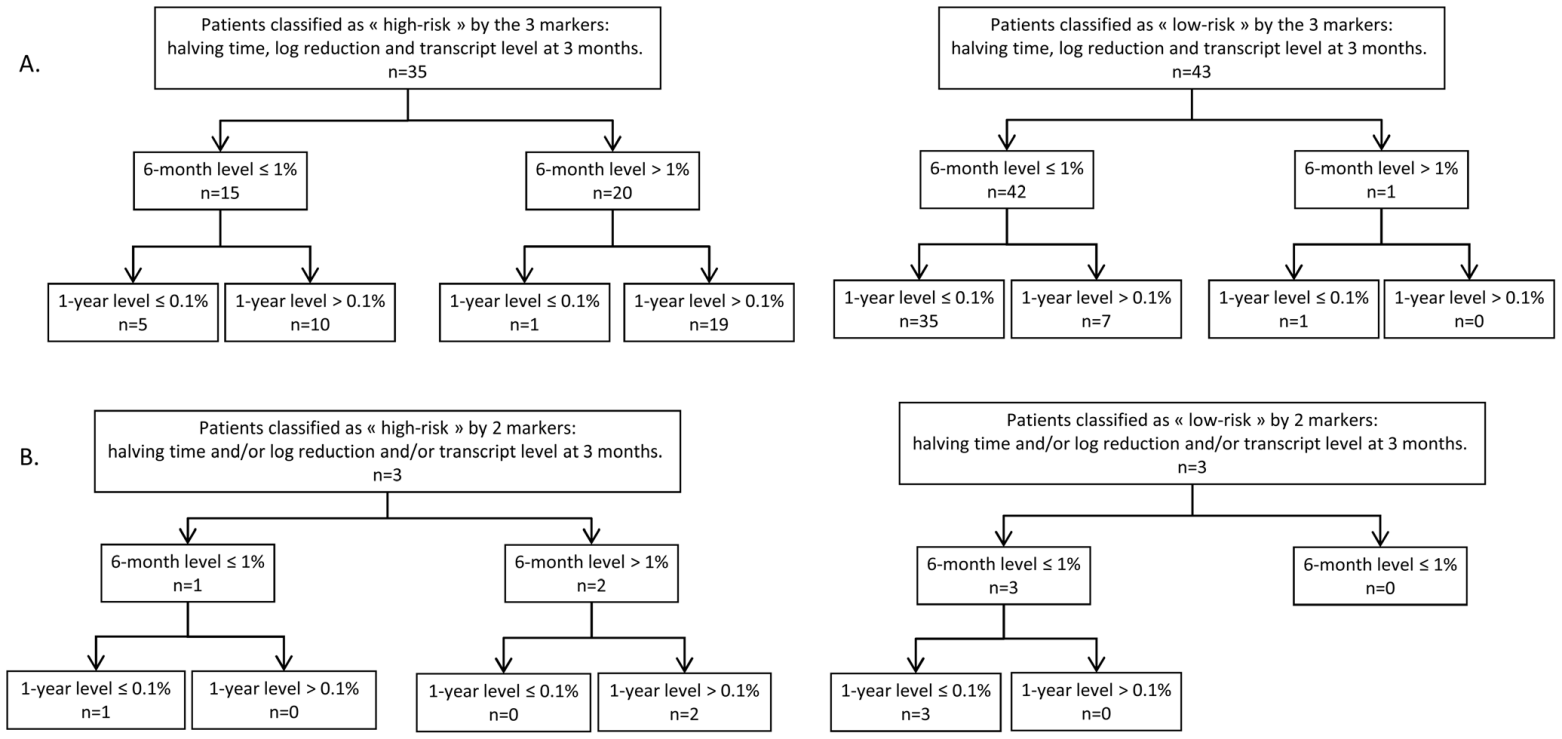
*BCR-ABL1/ABL1^IS^* transcript evolution of patients according to their classification with the 3 EMR markers. Three markers were used to assess early molecular response (EMR) with the use of *BCR-ABL1/ABL1^IS^* ratio: transcript level at 3 months, halving time and log reduction. Patients are classified as “high-risk” or “low-risk” according to the cut-offs described for each marker (see table 3) and their transcript level evolution at 6 and 12 months is reported. A: In most cases, all three markers were consistent to classify these patients as high risk (left part) or low risk (right part). B: In some discordant cases one of the markers showed a predictive value that differed from the other two. In this latter case, we took into account both concordant markers to classify the patients.

Therefore, the use of halving time, log reduction or transcript level at 3 months seem to have similar capacity to predict the molecular evolution of a patient, whatever CG is used. The comparison of EMR obtained according to the 3 markers using *ABL1* as CG is shown figure 6. Nevertheless, 7 patients still did not achieve MMR at 12 months although they obtained all the cut-offs listed here (*BCR-ABL1^IS^* ratio ≤0.99% at 3 and 6 months, a halving time ≤19 days and a log reduction <1.70) (7 patients out of 43: 16%). No treatment interruption or transformation was reported for these patients and all of them achieved the 0.1% threshold 15 to 22 months after treatment initiation without changing therapy (3 patients on Imatinib, 3 Dasatinib, 1 Nilotinib), suggesting that some patients may respond more slowly to TKI therapy. Conversely, 6 patients obtained MMR at 12 months although they were classified as high-risk patients according to each of these early molecular markers (*BCR-ABL1^IS^* ratio >0.99% at 3 months, a halving time >19 days and a log reduction <1.70) (6 patients out of 35: 17%). Interestingly, of these 6 patients, 5 had a *BCR-ABL1^IS^* ratio ≤1% at 6 months (3 on Imatinib, 1 Dasatinib, 1 Nilotinib) and 4 obtained a deep molecular response level of at least MR^4^ at a later stage. It is noteworthy that the cohort of discordant patients is too small to identify the best EMR marker (if one of them would be superior to others). Although all the markers seem to be relevant, the halving time cut-off identified allows the earliest detection of EMR failure.

### 5. Validation of the early 19 days monitoring time point

The observation that a halving time cut-off of 19 days may distinguish patients more likely to achieve MMR 1 year after diagnosis is a new finding that could change the current practices of molecular follow-up. In other words, a patient whose transcript level has not decreased by half after 19 days of treatment would benefit from an early switch of TKI. In order to confirm this finding, we evaluated this cut-off in a validation cohort of 58 patients. Only the *BCR-ABL1/ABL1* ratios were determined in this cohort, since we demonstrated the equivalence of both CG for the determination of EMR markers. The median halving time was 11 days (range, 3–171 days). MMR achievement rate was 70% in patients with a halving time ≤19 days (n = 43) and 7% in patients with a halving time >19 days (n = 15) (p<0.001), thus confirming the reliability of this cut-off. Similarly, cut-offs of 1.70 for log reduction and 0.99% for the 3-month ratio also showed a good distinction between patients achieving MMR and those who did not (log reduction: 89% vs 20%, p<0.001; 3–month ratio: 88% vs 27%, p<0.001).

### 6. Conclusions

#### Does *GUS* represent a better CG than *ABL1*?

Although *GUS* may be used as CG to quantify the *BCR-ABL1* transcript, our study highlights that the use of *BCR-ABL1/GUS* instead of *BCR-ABL1/ABL1* would have several consequences.

Firstly, *GUS* and *ABL1* are not expressed in the same range of values, which impacts the ratio *BCR-ABL1*/[CG] and requires a conversion factor to assess the connection between both measurements. This signifies that the use of *GUS* as CG would (i) imply a new standardization of the international scale and (ii) change in particular the MRD thresholds defined in the international recommendations to assess patient's response to therapy as the MMR threshold [1].

Secondly, we highlighted that *GUS* transcript levels at diagnosis show a direct correlation with *BCR-ABL1* levels, thus raising the hypothesis that *GUS* expression may depend on *BCR-ABL1* transcript level. Therefore, although *GUS* measurement is not affected by a technical quantification bias as observed with *ABL1*, this overexpression (directly linked to *BCR-ABL1* expression or not) does not allow more precise quantification of *BCR-ABL1* at diagnosis. Furthermore, *GUS* expression varies in a greater range of values than *ABL1* between the different disease times, which may preclude its use to assess the *BCR-ABL1* transcript kinetics of the EMR. Given the limitations described, we therefore do not consider that the replacement of *ABL1* by *GUS* represents an ideal option to assess EMR in CML patients.

#### Evaluation of molecular response with time-dependent variables

In the second part of our study, we investigated different ways recently described to assess the EMR and evaluated the ability of *BCR-ABL1/GUS* and *BCR-ABL1/ABL1* kinetics to predict the achievement of MMR 12 months after the TKI introduction. Using stringent inclusion criteria to ensure the reliability of our findings, we confirmed the strong predictive value of several early molecular markers in CML patients treated with 1^st^ or 2^nd^ generation TKI: the transcript level at 3 months of treatment, the halving time and the log-reduction of transcript levels between diagnosis and 3 months. We described the optimal cut-offs for the different markers for predicting 12-month MMR achievement in our cohort. In accordance with previous studies [3], [10], [12], we found that these molecular markers may predict patient molecular evolution with accuracy since the rate of molecular response was lower in patients with EMR failure than in patients who achieved EMR. Thus, in a near future the evaluation of the molecular response of each patient to TKI therapy will not only use the raw transcript levels but also different time-dependent variables assessing the transcript kinetics which are predictive of future molecular response and survival. Of note, there is currently no evidence that the use of *GUS* enables the prediction of patient evolution with more accuracy than *ABL1*. We identified a time point of 19 days after TKI introduction to assess whether halving time has been reached and these results were confirmed in an additional validation cohort. Patients whose transcript level has not decreased by half after 19 days of treatment are less likely to achieve MMR 1 year after diagnosis. It would therefore be appropriate to add this time point to patient monitoring in order to identify those patients likely to benefit from an early switch of TKI. Although these results remain to be validated on larger cohorts of patients, they suggest that it would be helpful to monitor *BCR-ABL1* levels at an earlier time point (close to 19 days) than realized in the current practice, in order to accurately assess response to TKI.
